# The predictive value of ^18^F-FDG PET-CT for assessing the clinical outcomes in locally advanced NSCLC patients after a new induction treatment: low-dose fractionated radiotherapy with concurrent chemotherapy

**DOI:** 10.1186/s13014-016-0737-0

**Published:** 2017-01-05

**Authors:** Maria Vittoria Mattoli, Mariangela Massaccesi, Alessandra Castelluccia, Valentina Scolozzi, Giovanna Mantini, Maria Lucia Calcagni

**Affiliations:** 1 Institute of Nuclear Medicine, Fondazione Policlinico Universitario Agostino Gemelli, Università Cattolica del Sacro Cuore, Largo A. Gemelli, 8, 00168 Rome, Italy; 2 Department of Radiation Oncology, Fondazione Policlinico Universitario Agostino Gemelli, Università Cattolica del Sacro Cuore, Largo A. Gemelli, 8, 00168 Rome, Italy

**Keywords:** ^18^F-FDG PET-CT, NSCLC, Chemo-radiotherapy, PERCIST, Tumour response

## Abstract

**Background:**

Patients with locally advanced non-small-cell lung cancer (LA-NSCLC) have poor prognosis despite several multimodal approaches. Recently, low-dose fractionated radiotherapy concurrent to the induction chemotherapy (IC-LDRT) has been proposed to further improve the effects of chemotherapy and prognosis. Until now, the predictive value of metabolic response after IC-LDRT has not yet been investigated. Aim: to evaluate whether the early metabolic response, assessed by ^18^F-fluoro-deoxyglucose positron emission-computed tomography (^18^F-FDG PET-CT), could predict the prognosis in LA-NSCLC patients treated with a multimodal approach, including IC-LDRT.

**Methods:**

Forty-four consecutive patients (35males, mean age: 66 ± 7.8 years) with stage IIIA/IIIB NSCLC were retrospectively evaluated. Forty-four patients underwent IC-LDRT (2 cycles of chemotherapy, 40 cGy twice daily), 26/44 neo-adjuvant chemo-radiotherapy (CCRT: 50.4Gy), and 20/44 surgery. ^18^F-FDG PET-CT was performed before (baseline), after IC-LDRT (early) and after CCRT (final), applying PET response criteria in solid tumours (PERCIST). Patients with complete/partial metabolic response were classified as responders; patients with stable/progressive disease as non-responders. Progression free survival (PFS) and overall survival (OS) were assessed using Kaplan-Meyer analysis; the relationship between clinical factors and survivals were assessed using uni-multivariate regression analysis.

**Results:**

Forty-four out of 44, 42/44 and 23/42 patients underwent baseline, early and final PET-CT, respectively. SUL_peak_ of primary tumour and lymph-node significantly (*p* = 0.004, *p* = 0.0002, respectively) decreased after IC-LDRT with a further reduction after CCRT (*p* = 0.0006, *p* = 0.02, respectively). At early PET-CT, 20/42 (47.6%) patients were classified as responders, 22/42 (52.3%) as non-responders. At final PET-CT, 19/23 patients were classified as responders (12 responders and 7 non-responders at early PET-CT), and 4/23 as non-responders (all non-responders at early PET-CT). Early responders had better PFS and OS than early non-responders (p ≤ 0.01). Early metabolic response was predictive factor for loco-regional, distant and global PFS (*p* = 0.02, *p* = 0.01, *p* = 0.005, respectively); surgery for loco-regional and global PFS (*p* = 0.03, *p* = 0.009, respectively).

**Conclusions:**

In LA-NSCLC patients, ^18^F-FDG metabolic response assessed after only two cycles of IC-LDRT predicts the prognosis. The early evaluation of metabolic changes could allow to personalize therapy. This multimodality approach, including both low-dose radiotherapy that increases the effects of induction chemotherapy, and surgery that removes the disease, improved clinical outcomes. Further prospective investigation of this new induction approach is warranted.

## Background

Lung cancer is the leading cause of death from cancer worldwide due to both its high incidence (estimated incidence of 1.8 million new cases in 2012) and fatality (overall ratio of mortality incidence of 0.87) (http://globocan.iarc.fr/Pages/fact_sheets_cancer.aspx?cancer=lung) [1]. Non-small-cell lung cancer (NSCLC) is the most frequent among lung cancers and, unfortunately, approximately 30% of patients are diagnosed with locally advanced NSCLC (LA-NSCLC), including unresectable stage II to III disease. Patients with LA-NSCLC, still represent a challenge for clinicians because the optimal treatment has not been clearly established and the prognosis is poor. To date, the Tumor-Node-Metastasis (TNM) staging system [2] - mainly based on morphologic and dimensional criteria and on anatomic localization - is considered the most important tool to define the disease stage, to guide treatment, and to estimate the prognosis. There is a general agreement among national and international guidelines that in patients with stage III NSCLC, the standard care is definitive concurrent chemo-radiotherapy (http://www.nccn.org/professionals/physician_gls/pdf/nscl.pdf) [3–7]. Despite the risk of severe toxicity of this therapeutic approach, and occasionally death from pneumonitis, less than 20% of the patients is free from disease progression after 5 years [8]. The prognosis, in addition to being poor, is variable because it differs among patients despite belonging to the same TNM stage (homogenous group); it is multifactorial because it may be influenced by environmental, patient and tumour factors, including molecular profile [9, 10]; it is dynamic because it may be different at staging, after treatment or at recurrence [11]. Therefore, aiming to improve the prognosis, the attention is focused on: 1) finding new therapeutic approaches, such as induction chemotherapy with or without concurrent low-dose radiotherapy before the standard treatment; 2) personalizing the therapy taking into account several factors, such as patient-related characteristics, tumour histology, co-morbidities; 3) assessing, as early as possible, the response to treatment. ^18^F-fluoro-deoxyglucose positron emission tomography (PET) is a well-established and useful tool to assess the metabolic response (early or late) [12, 13] and to select non-responder from responder patients allowing to tailor the treatment [14, 15], as well as to predict the outcome on the basis of early or late changes in tumour metabolism [16–18]. PET response criteria in solid tumours (PERCIST) [19] has been proposed in 2009 to evaluate the metabolic changes after treatment using the standardized uptake value normalized to lean body mass (SUL).

Until now, the predictive value of metabolic response after low-dose radiotherapy concurrent to the induction chemotherapy (IC-LDRT) in NSCLC patients has not yet been investigated. The aim of our study was to retrospectively evaluate whether the early metabolic response - assessed by ^18^F-fluoro-deoxyglucose positron emission-computed tomography (^18^F-FDG PET-CT) - could predict the disease progression free survival and the overall survival in patients with NSCLC stage III treated with a multimodal approach including IC-LDRT, neoadjuvant chemo-radiotherapy, and surgery.

## Methods

We retrospectively reviewed the clinical charts and electronic database of consecutive patients with histologically diagnosed NSCLC stage III, according to the 7th edition of the TNM classification for lung cancer [2], between January 2009 and October 2014. All patients, although judged medically fit for neo-adjuvant concurrent chemo-radiotherapy and for surgery, were either unresectable (due to N3 contralateral nodal involvement, mediastinal invasion, or bulky N2 disease) or resectable requiring a pneumonectomy.

Pulmonary physician evaluated the preoperative risk of mortality and long-term disability for major anatomic resection, according to Brunelli A et al. [20] performing cardiovascular evaluation and spirometry to measure the predicted post-operative Forced Expiratory Volume in 1 s (FEV_1_) and the diffusing capacity for carbon monoxide (DLCO). When considered appropriate, the following additional tests were performed: a low technology exercise test (stair climbing altitude -SCA- or shuttle walk distance -SWD), and a cardiopulmonary exercise test (peak oxygen consumption -VO_2_peak). In addition, quality-of-life was assessed using the Eastern Cooperative Oncology Group Performance Status (ECOG PS). Each patient of our cohort had ECOG PS less or equal to 1, and were classified as at *low risk* for anatomic surgical resection (FEV_1_ and DLCO >60%; FEV_1_ and DLCO within 30–60% *plus* SCA > 22 m or SWD > 400 m; FEV_1_ and DLCO within 30–60% *plus* SCA < 22 m or SWD < 400 m *plus* VO_2_peak >75%).

The staging evaluation included: total body diagnostic computed tomographic (CT), bone scintigraphy, brain CT or magnetic resonance (MR), and ^18^F-FDG PET-CT (baseline PET-CT). The pathologic proof of N2 and/or N3 involvement was required whenever lymph-nodes showed or the short axis higher than 1 cm on diagnostic CT or increased metabolic activity on ^18^F-FDG PET-CT. This retrospective study has been approved by the Ethics Committee of Fondazione Policlinico Universitario A. Gemelli, Rome.

### Treatment and follow-up

**Fig. 1:**
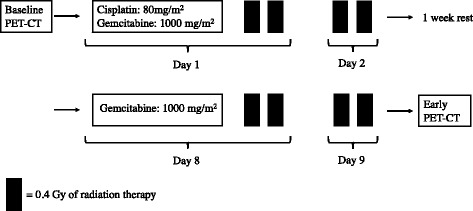
Treatment scheme of low-dose fractionated radiotherapy concurrent with induction chemotherapy. *Legend*: *Solid bars* represent 40 cGy of radiotherapy

The induction treatment protocol - consisted of two cycles of platinum-based chemotherapy - administered concurrently with “ultra-fractionated low dose” radiotherapy (LDRT, 40 cGy twice daily, days 1–2 and 8–9, every cycle) delivered with a conformal technique to the primary tumour, involved regional lymph-nodes and those adjacent, as showed in Fig. 1. After concurrent low-dose radiotherapy to induction chemotherapy (IC-LDRT), patients were re-evaluated and underwent: 1) surgery when medically fit patients showed a complete metabolic response on mediastinal lymph-nodes and/or resectable residual primary tumour extension; 2) neo-adjuvant concurrent chemo-radiotherapy (CCRT, total dose 50.4Gy, fractionation 1.8Gy/day) delivered with Linac using a conformal or intensity modulated technique to the sites of residual disease and, in case of mediastinal nodal clearance, originally involved nodal stations were also included in medically fit non-surgical patients without distant progression; 3) best supportive care, second-line chemotherapy, and/or palliative radiotherapy, according to the referring physician’s preference, in medically fit patients with distant progression and patients with poor medical conditions. After CCRT, patients were re-evaluated and underwent surgery or best supportive care, as reported above. Patients were followed every 3 months for 2 years with diagnostic total-body CT and brain MR or CT; then every 6 months indefinitely.

### ^18^F-FDG PET-CT acquisition protocol and response evaluation

Three ^18^F-FDG PET-CT were performed using the same acquisition and reconstruction protocols: before starting IC-LDRT (baseline PET-CT), at the end of IC-LDRT (early PET-CT), and at the end of CCRT (final PET-CT). The details of the study were explained and all patients provided written informed consent. All patients fasted for at least 6 h and presented a blood glucose level <150 mg/dl. PET-CT was performed 60 ± 10 min after administration of 240Mq of ^18^F-FDG (range: 185–333 MBq), according to the body mass index. No oral or intravenous contrast agents were administered nor bowel preparation were applied for patients. All the studies were performed using an integrated PET-CT device (3D Gemini GXL, Philips Healthcare, Cleveland, OH) with the same injected dose activity (±20%). An X-ray scout was carried to precisely define the spatial range of CT acquisition and a low-dose CT scan was performed from the base of the skull to the thighs (120 kV, 75 mA). CT images were used for the anatomical localization, for attenuation correction and fusion with PET images. Matched CT and PET images were reconstructed with a field-of-view of 50 cm. PET data were also shown in a rotating maximum intensity projection. PET and CT datasets were transferred to an independent computer workstation by DICOM (Digital Imaging and Communications in Medicine) transfer.

A semi-quantitative analysis was performed on PET-CT images using the Syntegra Philips fusion program by two nuclear medicine physicians (M.V.M. and V.S.) with PET-CT experience. PET Response Criteria in Solid Tumours (PERCIST) version 1.0 criteria [19] were used to evaluate the metabolic response on early and final PET-CT. According to the PERCIST criteria, the Standardized Uptake Value (SUV) corrected for lean body mass (SUL) was calculated [21]; the SUL_peak_ was determined using spheric regions of interest (with a diameter of about 1.2 cm) manually drawn over the primary tumour and over the lymph-node showing the highest ^18^F-FDG uptake. The percentage changes in SUL_peak_ (∆SUL_peak_) were also calculated between PET-CT scans. Patients with complete or partial metabolic response were classified as responder, and patients with stable or progressive disease as non-responders.

### Statistical considerations

The data were analysed by using the MedCalc Statistical Software version 12.7.2 with statistical significance set at *p* < 0.05. Results were reported with 95% Confidence Intervals (CI). Student’s paired *t* test was used to compare the SUL_peak_ at different time points. Disease progression free survivals (loco-regional, distant and global) and overall survival were calculated according to the Kaplan Meyer method and differences between groups were tested with the log-rank. Predictive factors for survivals were identified using univariate and multivariate regression analysis. Each factor whose *p* value was less than 0.1 in the univariate analysis was included in the multivariate analysis.

## Results

### Population and treatment

**Fig. 2:**
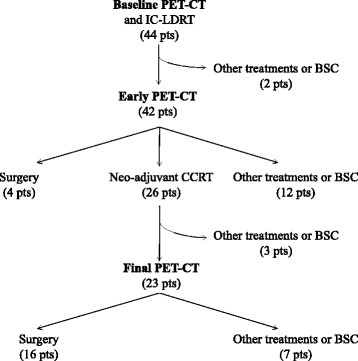
Treatment flow-chart. *Legend*: IC-LDRT: low-dose radiotherapy performed during induction chemotherapy; BSC: best supportive care; CCRT: concurrent chemo-radiotherapy; PET-CT: positron emission tomography-computed tomography

Forty-four patients (mean age: 66 ± 7.8 years, range: 47–81; 35 males) with NSCLC stage III were included in this analysis. The patients’ clinical characteristics are illustrated in Table 1. The majority of patients reported mediastinal lymph-node involvement of whom 26 with N2 (59.1%), and 10 with N3 (22.7%). Twenty-six out of 44 (59.1%) patients had stage IIIA, 18/44 (40.9%) patients stage IIIB. Figure 2 shows the treatment flow-chart. In particular: 44, 42 and 23 patients underwent baseline, early and final PET-CT, respectively.

**Table 1:** Patients’ characteristics

	N°	Percent
Histology		
Adenocarcinoma	25	56,8
Squamous cell carcinoma	15	34,1
Not otherwise specified	4	9,1
T classification		
1	3	6,8
2	15	34,1
3	15	34,1
4	11	25,0
N classification		
0	3	6,8
1	5	11,4
2	26	59,1
3	10	22,7
Clinical stage		
IIIA	26	59,1
IIIB	18	40,9
Total	44	100

All 44 patients completed the IC-LDRT and, subsequently, 4 (9.0%) underwent surgery, 26 (59.1%) underwent neo-adjuvant CCRT, 14 (31.8%) received palliative or best supportive care (in 3 patients the medical conditions worsened after IC-LDRT, 4 developed distant metastasis, and in 7 the CCRT was considered unsafe).

Twenty-six out of 44 patients (59.1%) underwent CCRT and, subsequently, 16/26 patients (61.5%) underwent surgery and 10/26 (38.5%) received palliative or best supportive care for the worsening of medical conditions or for the development of distant metastasis.

### ^18^F-FDG PET-CT

All 44 patients underwent baseline PET-CT (mean time from diagnosis: 6.6 ± 3.4 weeks): the mean value of SUL_peak_ of the primary tumour and lymph-node was 14.9 (±6.7) and 9.3 (±6.6), respectively.

**Fig. 3:**
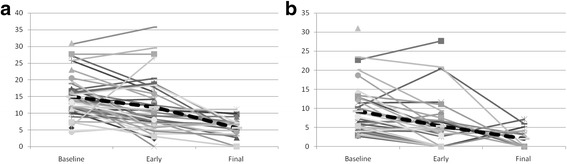
SUL_peak_ of primary tumour (**a**) and lymph node (**b**) in each patient at baseline PET-CT (*n* = 44), early PET-CT (*n* = 42), and final PET-CT (*n* = 23). *Legend*: Dashed lines represent mean values

Forty-two out of 44 patients (95.5%), who completed IC-LDRT, underwent early PET-CT (after a mean time of 4.5 ± 2.8 weeks): the mean value of SUL_peak_ of the primary tumour and lymph-node was 11.8 (±7.8) and 5.3 (±6.3), respectively. A significant reduction in SUL_peak_ of the primary tumour and lymph-node (*p* = 0.004, *p* = 0.0002), respectively was observed between baseline PET-CT and early PET-CT, as shown in Fig. 3. The mean value of ΔSUL_peak_ of the primary tumour and lymph-node between early PET-CT and baseline PET-CT was −14.9% (±63.5%) and −43.3% (±53.9%), respectively. No significant difference in tumour SUL_peak_ or lymph-node SUL_peak_ was found between stage IIIA and stage IIIB, either at baseline PET-CT or at early PET-CT.

Applying the PERCIST criteria at early PET-CT, 20/42 (47.6%) patients were classified as early responders of whom one with complete and 19 with partial metabolic response; 22/42 (52.3%) patients were classified as non-responders of whom 20 with stable disease and 2 with progressive disease. We describe, in detail, the treatment scheme performed after IC-LDRT and the follow-up of the 22 NR patients at early PET-CT. Twelve out of 22 NR patients underwent CCRT of whom: 7/12 showed local disease progression, 8/12 developed distant metastases, and 10/12 showed global disease progression. Eight out of 22 NR patients underwent palliative treatments, such as chemotherapy and/or palliative radiotherapy, of whom: 4 developed distant metastasis and 4 showed local disease progression. One out of 22 NR patient underwent surgery and developed distant metastasis. One out of 22 NR patient underwent best supportive care due to worsening of medical conditions.

At baseline PET-CT, responder patients did not show any significant difference in SUL_peak_ of the primary tumour and lymph-node when compared with non-responder patients.

Twenty-three out of 26 patients (88.5%) who completed CCRT underwent final PET-CT (after mean time 5.5 ± 1.7 weeks): the mean value of SUL_peak_ of the primary tumour and lymph-node was 5.6 (±2.8) and 1.8 (±2.2), respectively. A significant reduction (*p* = 0.0001, *p* = 0.0002, respectively) was observed in SUL_peak_ of the primary tumour and lymph-node between baseline PET-CT and final PET-CT, as well as between early PET-CT and final PET-CT (*p* = 0.0006, *p* = 0.02, respectively), as shown in Fig. 3.

Applying the PERCIST criteria at final PET-CT, 19/23 (82.6%) patients were classified as responders of whom two with complete and 17 with partial metabolic response; 4/23 (17.4%) patients were classified as non-responders all with stable disease. All patients classified as responders at early PET-CT remained responders at final PET-CT (12/12); 7/11 patients classified as non-responders at early PET-CT became responders at final PET-CT: 100% vs 63.6%, *p* = ns.

### Metabolic response and clinical outcomes

**Fig. 4:**
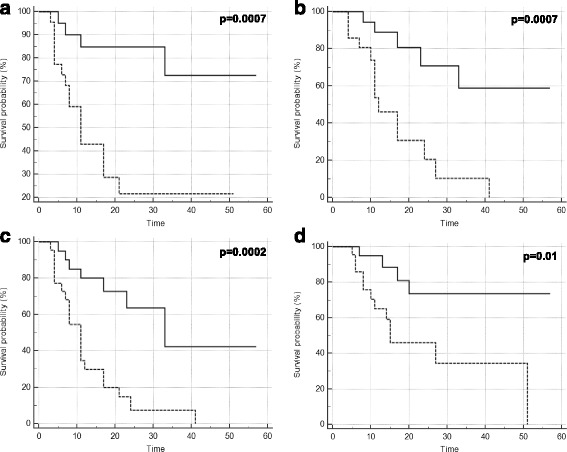
Loco-regional (**a**), distant (**b**) and global (**c**) progression free survival and overall survival (**d**) according to early metabolic response

In all patients (*n* = 44), the two-year loco-regional, distant and global disease progression free survival rates were 51.7, 48.3, and 34%, respectively; the two-year overall survival rate was 59%. The median loco-regional, distant, and global disease progression free survival times were 33, 24, and 17 months, respectively; the median overall survival time was 51 months. After IC-LDRT, responder patients at early PET-CT (20/42) had significant better loco-regional, distant, and global disease progression free survival and overall survival than non-responder patients (22/42): *p* = 0.0007, *p* = 0.0007, *p* = 0.0002, *p* = 0.01 (Fig. 4, Table 2).

**Table 2:** Two-year survival endpoints according to metabolic response at early PET-CT in different patients’ groups

	N°	Early metabolic response		Loco-regional PFS		Distant PFS		Global PFS		Overal survival	
Patients who underwent early PET-CT	42	R	20	84.7%	*p* = 0.0007	70.7%	*p* = 0.0007	63.6%	*p* = 0.0002	73.5%	*p* = 0.01
		NR	22	21.5%		20.5%		7.4%		45.9%	
Patients who underwent final PET-CT	23	R	12	100%	*p* = 0.007	67.9%	*p* = 0.03	67.9%	*p* = 0.02	77.8%	*p* = 0.06
		NR	11	35.4%		30.5%		15.2%		62.3%	
Patients who underwent surgery	20	R	13	100%	*p* = 0.04	83.3%	*p* = 0.01	83.3%	*p* = 0.04	87.5%	*p* = 0.16
		NR	7	41.7%		42.9%		28.6%		66.7%	

*R* responder, *NR* non-responder, *PFS* progression free survival, *PET-CT* positron emission tomography-computed tomography

In patients who underwent CCRT (*n* = 26), the two-year loco-regional, distant, and global progression disease free survival rates were 67.3, 54.6, 44.7%, respectively; the two-year overall survival rate was 65.4%. Regarding patients who underwent baseline, early and final PET-CT (*n* = 23), patients classified as responders at early PET-CT (*n* = 12/23) had significant better loco-regional, distant, and global progression disease free survival than patients classified as non-responder at early PET-CT (11/23): *p* = 0.007, *p* = 0.03, *p* = 0.02, respectively (Tables 2).

In patients who underwent surgery (*n* = 20), the two-year loco-regional, distant and global disease progression free survival rates were 77.3, 67.5, and 59.8%, respectively; the two-year overall survival rate was 78.6%. Also in this sub-group, patients classified as responders at early PET-CT (*n* = 13) had significant better loco-regional, distant and global disease progression free survival than non-responder patients (*n* = 7): *p* = 0.04, *p* = 0.01, *p* = 0.04, respectively (Table 2).

At univariate analysis, surgery, SUL_peak_ of N at staging, and metabolic response evaluated at early PET-CT were significant predictive factors for loco-regional disease progression free survival (*p* = 0.0001, *p* = 0.02, *p* = 0.001, respectively); SUL_peak_ of T at staging and metabolic response evaluated at early PET-CT were significant predictive factors for distant disease progression free survival (*p* = 0.02, *p* = 0.01, respectively); surgery and metabolic response evaluated at early PET-CT were significant predictive factors for global disease progression free survival (*p* = 0.0006, *p* = 0.0002, respectively); surgery and metabolic response evaluated at early PET-CT were significant predictive factors for overall survival (*p* = 0.02, *p* = 0.02, respectively). The multivariate analysis showed, surgery and metabolic response at early PET-CT were significant predictive factors for loco-regional disease progression free survival (*p* = 0.03, *p* = 0.02, respectively); SUL_peak_ of T at staging and metabolic response at early PET-CT were significant predictive factors for distant disease progression free survival (*p* = 0.04, *p* = 0.01, respectively); age, surgery and metabolic response at early PET-CT were significant predictive factors for global disease progression free survival (*p* = 0.04, *p* = 0.009, *p* = 0.005, respectively). No parameter was a significant predictive factor for overall survival (Table 3).

**Table 3:** Multivariate analysis for potential prognostic factors

	Loco-regional PFS			Distant PFS			Global PFS			Overall Survival		
Characteristics	OR	95% CI	P-value	OR	95% CI	P-value	OR	95% CI	P-value	OR	95% CI	P-value
Gender	0,41	0,03–4,94	0,4804	1,16	0,15–8,71	0,8880	0,80	0,09–6,83	0,8367	4,31	0,56–32,84	0,1591
Age	1,02	0,90–1,15	0,7026	0,92	0,83–1,02	0,1201	0,88	0,78–0,99	0,0413	1,04	0,93–1,15	0,4658
Baseline SUL_peak_ of T				0,87	0,75–0,99	0,0489						
Baseline SUL_peak_ of N	1,16	0,97–1,38	0,0984									
Early metabolic response	0,14	0,02–0,82	0,0292	0,15	0,03–0,71	0,0171	0,05	0,00–0,41	0,0059	0,24	0,05–1,06	0,0608
Surgery	0,14	0,02–0,91	0,0399				0,04	0,00–0,44	0,0093	0,23	0,03–1,39	0,1093

*SUL*_*peak*_ standardized uptake value corrected for lean body mass, *PFS* progression free survival, *OR* Odds ratio, *CI* confidence interval, *T* primary tumour, *N* lymph-node

## Discussion

To the best of our knowledge, this is the first study that has evaluated the role of ^18^F-FDG PET-CT performed early after low-dose fractionated radiotherapy concurrent to the induction chemotherapy for predicting the clinical outcomes in patients with non-small-cell lung cancer stage III. In these patients, several efforts have been made to find the best therapeutic approach to improve the prognosis. The National Comprehensive Cancer Network guidelines (NCCN) (http://www.nccn.org/professionals/physician_gls/pdf/nscl.pdf) recommend concurrent definitive chemo-radiotherapy as the standard treatment in patients with locally advanced disease. However, in patients with ipsilateral mediastinal or sub-carinal lymph-node disease (N2), the induction chemotherapy with or without concurrent radiotherapy, is an additional option to reinforce the effects of neo-adjuvant chemo-radiotherapy aiming to downstage the disease. In N2-patients, the choice between concurrent definitive chemo-radiotherapy and induction therapy is based on factors regarding either the patient or the neoplastic disease: clinical conditions, primary tumour resectability, and extension and bulkiness of mediastinal lymphadenopathy [22]. Despite these different approaches, the patients have a slight chance of survival or being free from disease long-term progression. In particular, in patients treated with concurrent chemo-radiotherapy the predominant failure is the loco-regional recurrence ranging from 63 to 84% at three years [23]. From these data, it is possible to state that the current radiation dose seems insufficient to reliably establish the local control. A randomized study (the Intergroup 0139) [24] in patients with NSCLC stage IIIA (N2) has demonstrated that, by completely removing the tumour and the lymph-node metastasis, surgery improves the local control when compared with radiotherapy, however, not improving the overall survival. Therefore, it is still debated whether surgery with comorbidity risk is a justifiable option in patients with such an aggressive and unfavourable disease. In this scenario, it is of paramount importance to have a reliable tool to evaluate the response to treatment as early as possible, in order to prompt select patients who either continue, or change or intensify the treatment, personalizing it. ^18^F-FDG PET-CT is largely used in oncology particularly to monitor the early changes in glucose metabolism after treatment and for prognostic information [12–18]. The PERCIST criteria were proposed as a functional method to evaluate the treatment response in several cancers [19].

From our results, we observed that at baseline PET-CT, the glucose avidity of primary tumours and lymph-nodes, as expressed by SUL_peak_, was high and variable among patients and similar to that reported by Ding et al. [25]. The enhanced trapping and the large variability of ^18^F-FDG in the tumour cells is still being studied because several biological mechanisms, such as the up-regulation of glucose transporters and hexokinase enzymes, tumour aggressiveness, hypoxia, etc. [26–29] are responsible for the different levels of ^18^F-FDG uptake, as well as the histotype and the histological grading. In our population, the predominant histotype was adenocarcinoma, as expected being the most frequent histotype [30], that has a relatively low ^18^F-FDG avidity [31, 32]; on the other hand, also the squamous histotype was well-represented that has a high ^18^F-FDG avidity [33, 34]. In addition, in both histotypes the histological grading was G2 or G3 (moderately or poorly differentiated) that are typically more ^18^F-FDG avid than G1 (well-differentiated) [33–36]. Moreover, we did not find any significant difference in metabolic activity between stage IIIA and stage IIIB. This result can be explained because this classification is based on two morphological criteria (size of primary tumour and anatomic localization of lymph-node) and it does not take into account any metabolic characteristic (^18^F-FDG uptake/SUV) that, conversely, reflects biological features of tumour cells.

In the last years, low-dose fractionated radiotherapy (<1 Gy) concurrent to the induction chemotherapy has been proposed to further improve the effects of induction chemotherapy in several solid tumours, such as locally advanced breast cancer [37, 38], pancreatic cancer [39], head neck carcinoma [40], glioblastoma [41] and NSCLC [42]. In-vitro studies have demonstrated that LDRT chemo-potentiates the effects of cisplatin [42], and that concomitant four low-dose radiotherapy fractions provide the optimal cell killing, either with apoptosis or clonogenic inhibition [43, 44], without further increasing toxicity. Moreover, in-vivo studies on several types of epithelial cancer have demonstrated that LDRT is feasible and well tolerated [42], having an overall response rate of 45% [42, 45]. In our study, after two cycles of IC-LDRT, we observed a significant reduction of the metabolic activity, as expressed by the SUL_peak_ reduction, indicating that also this approach acts on all tumour cells both in primary tumours and lymph-nodes. In particular, the metabolic reduction was more evident in lymph-nodes: this finding could be probably due to their higher radio-sensitivity that could be linked either to a higher cell replication rate or to their less amount of hypoxia and/or necrosis in lesions with small size [46]. In addition, after IC-LDRT, approximately 50% of patients showed good results, such as complete and/or partial metabolic response and, therefore, defined as responders. This finding allows to state that also this induction approach, considered preparatory for the stronger subsequent therapies, acts on tumour cells killing or stunning them [42, 43] as demonstrated by the reduction of metabolic activity. These favourable results allowed modifying, although in few patients, the planned therapeutic scheme leading them directly to surgery. Regarding non-responder patients, ^18^F-FDG PET-CT allowed to identify not only patients with stable disease but also those who developed earlier distant metastasis. In our study, the rate of patients with progressive metabolic disease after IC-LDRT was remarkably lower than that recently reported in literature [47] in a similar population treated with induction chemotherapy and assessed with PERCIST criteria. The addition of low-dose radiotherapy to the induction chemotherapy, probably induces an early improvement of anti-tumour immune-response also against micrometastases outside of the radiation field [48].

We did not find any significant difference in SUL_peak_ either in primary tumour or in lymph-node by analysing the baseline PET-CT in responder and in non-responder patients. Since the metabolic activity of the primary tumour and/or neoplastic lymph-nodes at baseline PET-CT did not distinguish responders from non-responders, and considering that tumours are inhomogeneous, it is hoped that SUL_peak_ limitation could be overcome by using either more sophisticated parameters (Ki, k1, k2, ecc) obtained by absolute quantification, or evaluating additional metabolic pathways (hypossia, aminoacids, ecc.) with other radiotracers. These new approaches could highlight differences between responders and non-responders at baseline PET-CT, still hidden at SUL_peak.._

Lastly, in patients that completed the treatment (IC-LDRT plus CCRT), we found a further reduction of ^18^F-FDG uptake at final PET-CT, as expected, in primary tumours and lymph-nodes when compared either with baseline PET-CT or early PET-CT, suggesting a further effect of the CCRT on tumour cells. The completed treatment provided very good results: while all responder patients at early PET-CT persisted as responder at final PET-CT, the CCRT allowed to rescue several patients defined as non-responders after IC-LDRT increasing the rate of responder patients at final PET-CT.

In our population, we found good overall survival: this result is in line with that reported by some Authors [49, 50] in patients treated with standard chemo-radiotherapy and surgery but remarkably higher than that reported by other Authors [47, 51] in patients treated with induction chemotherapy. These data further suggest that the improvement of the overall survival seems to be influenced by: 1) surgery, that removing the disease, can play an important role in patients with locally advanced NSCLC; 2) induction therapy, particularly when concurrent low-dose radiotherapy is added, that boosts the subsequent treatment; 3) the personalization of the treatment that allows to tailor the therapy to the individual patient. Finally, in our population we observed that the rate of loco-regional recurrence was less than 30% with a tendency to decrease when loco-regional treatments, in particular surgery, were performed allowing to achieve a more effective loco-regional control.

Regarding the relationship between clinical outcomes and metabolic response, we observed that patients classified as non-responders at early PET-CT had a shorter loco-regional, distant, and global disease progression free survival, and overall survival than those classified as responders. This result was also observed in patients that completed all treatment (IC-LDRT, CCRT) and in those who underwent surgery. Therefore, we can assert that the early metabolic response performed after two cycles of IC-LDRT using “functional” PERCIST criteria, allows to identify patients with poor prognosis: indeed, the majority of patients classified as non-responders showed early disease progression during follow-up. From a clinical point of view, non-responder patients after IC-LDRT despite becoming responders after CCRT and/or surgery, have poor prognosis: therefore, the choice of a more appropriate therapy after IC-LDRT still represents a difficult challenge. Probably for these patients even more intensified local treatment are needed.

Similarly to our study, Fledelius J et al. [47] applied PERCIST criteria to retrospectively evaluate the prognostic value of PET-CT after induction chemotherapy. Although the metabolic response rate appears similar (almost 50%) between the two studies, the clinical outcomes were different: both our responder and non-responder patients had remarkable longer disease progression free survival and overall survival. This finding could be due to differences in baseline features of the included patients or to a potential effect of low-dose radiation therapy as a chemo-enhancer [42] to the subsequent concurrent radio-chemotherapy or to the “tailored” treatment scheme for the individual patients.

From our data, even if only at a univariate analysis, we found that the distant and the loco-regional progression free survival were affected by SUL_peak_ of the primary tumour and SUL_peak_ of the lymph-nodes at staging, respectively: higher SUL_peak_, higher aggressiveness, higher chances to develop distant micrometastasis, as well as loco-regional recurrences. The multivariate analysis showed that surgery was a predictive factor for assessing the loco-regional and global disease progression free survival, suggesting that medically fit patients could benefit from surgery improving the disease control. Moreover, the early metabolic response was the only predictive factor for assessing all disease endpoints: it is well known that the tumour metabolism, as expressed by SUL_peak_, reflects the metabolic behaviour in terms of cell aggressiveness, proliferation, and de-differentiation [52, 53]. Therefore, the early SUL_peak_ reduction suggests that the tumour cells are either more sensitive to treatment or, probably, not so aggressive, despite their high metabolic activity at staging. From a clinical point of view, the early metabolic response, being predictive of prognosis, allows to personalize the subsequent therapeutic strategy, taking into account the functional changes in addition to the clinical conditions and the morphological aspects. Lastly, any parameter was able to predict the overall survival: NSCLC stage III still remains a shadow area for clinicians and further efforts should be made.

The main limitations of our study are: the relatively small size of the population and its retrospective characteristics, however a series of consecutive patients with LA-NSCLC were included and clinical data were prospectively collected on an electronic data-base.

## Conclusions

^18^F-FDG PET-CT is a reliable tool to: assess the metabolic response also in LA-NSCLC patients after low-dose radiotherapy concurrent to the induction chemotherapy; early select non-responder from responder patients allowing to tailor the subsequent therapeutic approach; predict the clinical outcomes on the basis of early metabolic changes. Indeed, patients with LA-NSCLC are a heterogeneous group in terms of tumour volume/extension, lymph-nodal spread and prognosis; therefore, the important functional information provided earlier by ^18^F-FDG PET-CT could allow to select different subgroups of patients that may deserve different therapeutic strategies, beyond TNM staging based on morpho-dimensional criteria and anatomic localization. Moreover, this multimodal approach, including both the low-dose radiotherapy that increases the effect of induction chemotherapy, and surgery that removes the disease, has proven to be a promising treatment option, improving the clinical outcomes in patients with such an aggressive and unfavourable disease. Further randomized and controlled prospective investigations of this new induction strategy are warranted.

## Abbreviations

^18^F-FDG PET-CT: ^18^F-fluoro-deoxyglucose positron emission-computed tomography
CCRT: Concurrent chemo-radiotherapy
IC-LDRT: Concurrent low-dose radiotherapy to induction chemotherapy
LA-NSCLC: Locally advanced non-small-cell lung cancer
PERCIST: PET response criteria in solid tumours
SUL: SUV corrected for lean body mass
SUV: Standardized uptake value
TNM: Tumor-Node-Metastasis staging system
